# Supplementary material for: Mechanisms governing the pioneering and redistribution capabilities of the non-classical pioneer PU.1
Source: Nat Commun. 2020 Jan 21;11:402. doi: 10.1038/s41467-019-13960-2 (PMC6972792; doi:10.1038/s41467-019-13960-2)
Supplement: Supplementary file 7 — Source data [file 41467_2019_13960_MOESM7_ESM.zip › Source_Data/FigureS2/FigureS2B_MotifScanOutput/KmeansCluster8/homerResults/motif9.similar.html]

motif9

## Information for motif9

T
C
G
A
C
T
G
A
A
T
G
C
C
A
G
T
A
C
G
T
A
G
T
C
A
G
T
C
G
A
C
T
A
G
T
C
C
G
T
A
T
C
A
G
C
T
G
A
T
C
A
G
  
Reverse Opposite:  

A
G
T
C
A
G
C
T
A
G
T
C
C
G
A
T
T
C
A
G
C
T
G
A
C
T
A
G
A
C
T
G
T
G
C
A
G
C
T
A
T
A
C
G
G
A
C
T
A
C
G
T
  

|  |  |
| --- | --- |
| p-value: | 1e-44 |
| log p-value: | -1.025e+02 |
| Information Content per bp: | 1.655 |
| Number of Target Sequences with motif | 154.0 |
| Percentage of Target Sequences with motif | 10.19% |
| Number of Background Sequences with motif | 1132.6 |
| Percentage of Background Sequences with motif | 2.49% |
| Average Position of motif in Targets | 98.6 +/- 50.2bp |
| Average Position of motif in Background | 101.0 +/- 74.3bp |
| Strand Bias (log2 ratio + to - strand density) | 0.1 |
| Multiplicity (# of sites on avg that occur together) | 1.03 |
| Motif File: | file (matrix) reverse opposite |

### Similar de novo motifs found

|  |  |  |  |  |  |  |  |
| --- | --- | --- | --- | --- | --- | --- | --- |
| Rank | Match Score | Redundant Motif | P-value | log P-value | % of Targets | % of Background | Motif file |
| 1 | 0.802 | C T A G A C G T C A G T T G A C G T A C G A C T G A T C C T G A G T C A T C G A | 1e-21 | -50.652043 | 2.78% | 0.34% | motif file (matrix) |
